# A novel capsular operon and potentially conjugative plasmids in extensively drug-resistant urogenital *Haemophilus parainfluenzae*

**DOI:** 10.3389/fmicb.2025.1659538

**Published:** 2025-10-08

**Authors:** Lucía Saiz-Escobedo, Mónica Ballestero-Tellez, Mireia Rajadell, Marc Garreta-Esteban, Laura Gisbert, Irene Cadenas-Jiménez, Rosa Maria Costa, Fe Tubau, David Sánchez-Ramos, M. Angeles Domínguez, Kyriaki Xanthopoulou, Paul G. Higgins, Carmen Ardanuy, Aida González-Díaz, Sara Marti

**Affiliations:** ^1^Microbiology Department, Hospital Universitari de Bellvitge, IDIBELL-UB, Barcelona, Spain; ^2^Department of Pathology and Experimental Therapeutics, University of Barcelona, Barcelona, Spain; ^3^Clinical Microbiology Department, Catlab, Barcelona, Spain; ^4^Infectious Diseases Department, Hospital Universitario Mutua Terrassa, Terrassa, Spain; ^5^Research Network for Respiratory Diseases (CIBERES), ISCIII, Madrid, Spain; ^6^Research Network for Infectious Diseases (CIBERINFEC), ISCIII, Madrid, Spain; ^7^Institute for Medical Microbiology, Immunology and Hygiene, Faculty of Medicine and University Hospital Cologne, University of Cologne, Cologne, Germany; ^8^German Center for Infection Research, Partner Site Bonn-Cologne, Cologne, Germany; ^9^Department of Medicine, School of Medicine, University of Barcelona, Barcelona, Spain

**Keywords:** *Haemophilus parainfluenzae*, capsular operon, antimicrobial resistance, conjugative plasmid, whole-genome sequencing, extended-spectrum β-lactamase, STIs, genomic surveillance

## Abstract

**Introduction:**

*Haemophilus parainfluenzae* is an opportunistic pathogen increasingly associated with urogenital infections and multidrug resistance. While polysaccharide capsules are known virulence factors in *H. influenzae*, their role in *H. parainfluenzae* remains poorly understood. This study aimed to characterize a new capsular operon identified in *H. parainfluenzae* and analyze the antimicrobial resistance profile of strains carrying this operon to provide insights into their pathogenic potential.

**Methods:**

Two clinical isolates from genital ulcers were subjected to whole-genome sequencing. The capsular operon was identified and characterized using comparative genomics. Antimicrobial susceptibility was determined using broth microdilution, and the resistance determinants were identified through genomic analysis.

**Results:**

A novel capsular operon, designated *H. parainfluenzae* HPAR_type4, was identified in both isolates. This operon spans 11,885 bp and comprises nine predicted open reading frames (ORFs) organized into the three regions characteristic of *Haemophilus* spp. Regions I, III, and the first ORF of region II showed high homology with the HPAR_type1 operon, while the remaining ORFs in region II shared identity with the *cpsB* and *cpsC* genes from *H. sputorum* HSPU_type1. Both strains exhibited multidrug resistance, with one strain carrying a CTX-M-15 extended-spectrum β-lactamase gene integrated in an integrative and conjugative element (ICE), ICE*Hpa*HUB6. Two distinct potentially conjugative plasmids were identified, each carrying genes related to replication, mobility, and putative virulence functions.

**Conclusion:**

The identification of a novel capsular operon in multidrug-resistant *H. parainfluenzae* strains highlights the species genetic plasticity and potential for increased virulence. These findings underscore the importance of ongoing surveillance in clinical settings to monitor the spread of antibiotic resistance and novel virulence factors, which may contribute to its pathogenicity and impact future treatment strategies.

## 1 Introduction

*Haemophilus parainfluenzae* is an opportunistic Gram-negative pathogen that colonizes the respiratory and urogenital tracts (Nørskov-Lauritsen, 2014). This species has been implicated as a cause for various diseases, including respiratory tract infections (Kosikowska et al., 2016), endocarditis (Revest et al., 2016) and arthritis (Baron et al., 2020), with a notably high prevalence as a cause of urethritis in recent years (Ducours et al., 2020; Hsu et al., 2015; Magdaleno-Tapial et al., 2019).

Polysaccharide capsules, a major virulence factor in *H. influenzae*, have also been identified in *H. parainfluenzae*, although their precise role in this species remains unclear. In *H. influenzae*, these capsules are crucial for evading host immune responses and have been used in vaccine development (González-Díaz et al., 2019; Sierra et al., 2021; Watts and Holta, 2019). The genetic blueprint for these polysaccharide capsules in the genus *Haemophilus* is encoded in the *cap* locus, which comprises three different regions. Region I has four conserved genes (*bexABCD*) that encode the export apparatus for the translocation of capsular polysaccharides. Region II contains serotype-specific genes (three to eight genes) involved in the polysaccharide biosynthesis. Finally, region III has two genes (*hcsAB*) responsible for polysaccharide transport across the outer membrane (Potts et al., 2019). To date, three different capsular operons have been described in *H. parainfluenzae*: HPAR-type1 (González-Díaz et al., 2019) and HPAR-type2 (Sierra et al., 2021), both detected in urogenital strains, and the serotype f-like operon recently identified in colonizing strains from nasopharyngeal samples of healthy children in Portugal (Bajanca-Lavado et al., 2022).

Along with virulence factors, antimicrobial resistance has emerged as a significant concern in *H. parainfluenzae*. The increasing incidence of multidrug resistance in this species is attributed to target-gene mutations and the acquisition of transferable resistance genes. Extensively drug-resistant (XDR) strains have been detected in recent years, particularly in urogenital samples, showing resistance to β-lactams, fluoroquinolones, macrolides, chloramphenicol, trimethoprim-sulfamethoxazole and tetracycline. Notably, our group previously reported the presence of a CTX-M-15 extended spectrum β-lactamase (ESBL) in four XDR *H. parainfluenzae* strains isolated from three urethral exudates and a genital ulcer (Saiz-Escobedo et al., 2023). This finding was subsequently corroborated by the identification of the CTX-M-15 ESBL in a urogenital XDR *H. parainfluenzae* strain from France (Caméléna et al., 2024).

The main objective of this study is to characterize a novel capsular operon detected in two XDR *H. parainfluenzae* strains isolated from genital ulcers in MSM patients. Additionally, we also aim to provide a comprehensive genotypic and phenotypic characterization of these capsulated strains, with particular emphasis on their antimicrobial resistance profiles.

## 2 Materials and methods

### 2.1 Bacterial strains and antimicrobial susceptibility

Two *H. parainfluenzae* strains harboring a new capsular operon, identified by whole genome sequencing (WGS), were included in this study. The first strain, HUB-HP17268, was isolated in 2022 from a genital chancre exudate of a young male patient with a genital ulcer who attended the Hospital Universitari de Bellvitge (HUB) in Barcelona (Cataluña, Spain). The second strain, HUMT-HP05, was isolated in 2024 from a genital ulcer of a young MSM patient attending the Pre-exposure Prophylaxis (PrEP) unit at Hospital Universitari Mútua de Terrassa (HUMT), also in Cataluña, Spain.

Strains were cultured on chocolate agar (BioMérieux, Marcy-l’Étoile, France) and incubated at 37 °C in a 5% CO_2_ atmosphere. Bacterial identification was performed by MALDI-TOF mass spectrometry (Bruker and MS-Prime BioMérieux).

Antimicrobial susceptibility was assessed by microdilution using STRHAE2 Sensititre commercial panels (Thermo Fisher Scientific, Waltham, MA, USA). Double-disk synergy test was conducted to detect ESBL production, using amoxicillin-clavulanic acid along with the β-lactams, cefotaxime, ceftazidime, aztreonam, and cefuroxime. All susceptibility testing procedures followed the European Committee on Antimicrobial Susceptibility Testing criteria for *H. influenzae*.^1^

### 2.2 Whole genome sequencing

Genomic DNA was extracted using the QIAmp DNA mini kit (Qiagen) and quantified with Qubit 4 (Thermo Fisher Scientific). Short-read libraries were prepared using the Illumina DNA Prep kit for paired-end sequencing (2 × 300 bp) on the MiSeq Platform (Illumina Inc.). Long-read libraries were prepared with Native Barcoding Expansion (EXP-NBD196) and Ligation Sequencing kit (SQK-LSK109), followed by sequencing on FLOMIN106D flow cells (R9.4.1) from Oxford Nanopore Technologies. Reads were assembled using Bactopia^2^ and hybrid assemblies combining short and long reads were generated using the Unycicler pipeline^3^ (Wick et al., 2017). Raw reads were uploaded to the European Nucleotide Archive (PRJEB88731). To elucidate genomic similarity between both genomes, a whole genome alignment was performed with Snippy v4.6.0^4^ using HUMT-HP05 as reference. The final alignment was screened with Geneious R9 (version 9.1.7, Biomatters) and recombinant blocks were defined as the presence of 3 SNPs in windows of 100–1,000 bp.

### 2.3 Capsular operon determination

Capsular loci were identified *in silico* using HiCap^5^ (Watts and Holta, 2019). BLASTn searches were performed with Geneious R9 (version 9.1.7, Biomatters) to compare the sequences against known capsular operons from *Haemophilus* spp. Reference sequences included *H. parainfluenzae* HPAR-type1 (MH644108), HPAR-type2 (MT185932), and serotype f-like operon (JAMLEO010000002:224525-240017); *H. influenzae* serotype a (CP017811:328547-341597), serotype b (NC_016809:774911-790627), serotype c (HQ651151), serotype d (HM770877), serotype e (FM882247) and serotype f (CP005967:675016-687441); *H. sputorum* HSPU_type1 (NZ_AFNK01000031:141496-153330) and HSPU_type2 (QEPN01000003:47115-64856); and *H. haemolyticus* (SDPB01000019:33146-48666). Open reading frames (ORFs) were predicted, and sequence identity between putative genes/proteins from different capsular operons was calculated as the percentage of identical number of bases/residues divided by the length of the longest gene/protein.

### 2.4 Resistance determinants and integrative and conjugative elements (ICE)

Acquired resistance genes were screened using the AMRFinder+ v3.11.18.^6^ Resistance-associated mutations and mobile genetic elements were analyzed with Geneious R9 using *H. parainfluenzae* T3T1 (NC_015964) as a reference genome. ICE structures were examined using ICE*HpaHUB6* and ICE*HpaHUB7* as references (Saiz-Escobedo et al., 2023).

### 2.5 Plasmid description

Potential plasmids were screened with the PlasmidFinder tool^7^ of the Center for Genomic Epidemiology. Plasmid sequences were annotated using PROKKA^8^ to predict ORFs and functional elements. Homologous sequences were identified by BLASTx and BLASTn searches against public databases. Plasmid structures and virulence genes were analyzed and curated using Geneious R9 (version 9.1.7, Biomatters). For graphical representation, plasmid structures were visualized with Geneious R9, GC content was represented using Proksee (proksee.ca), and the final figure was refined with Inkscape.^9^

### 2.6 Ethics

This study complied with the Declaration of Helsinki principles from the World Medical Association and was approved by the Clinical Research Ethics Committee of Bellvitge University Hospital (PR075/21). Written informed consent was not required as this was a retrospective observational study with isolates obtained as part of routine microbiological testing. Patient confidentiality was maintained by anonymizing all personal data in accordance with Spanish legal regulations (LOPD 15/1999 and RD 1720/2007). Biological sample management adhered to Law 14/2007 on Biomedical Research.

## 3 Results

### 3.1 Clinical strains and patient background

HUB-HP17268 and HUMT-HP05 were isolated from genital ulcer swabs taken from two young men with multiple previous STIs, including chlamydia, gonorrhea, syphilis, and genital mycoplasma. Both patients lacked symptoms such as abdominal pain, diarrhea, anal bleeding, dysuria, or urogenital discharge and consulted for the mentioned genital ulcers.

**Patient 1 (HUB):** 34-year-old MSM with positive serology for *Treponema pallidum* (RPR titer of 1:8). HIV-positive undergoing antiretroviral therapy. In the current episode, the patient tested positive by PCR for *Chlamydia trachomatis* and *Mycoplasma genitalium* and was treated with a combination of intramuscular ceftriaxone and oral doxycycline.

**Patient 2 (HUMT):** 28-year-old MSM with positive serology for *T. pallidum* (RPR titer of 1:16), with adenopathy and a history of multiple STIs. This patient had been enrolled in a pre-exposure prophylaxis (PrEP) program since May 2022. In the 6 months preceding this episode, he received intramuscular ceftriaxone for gonococcal urethritis. In this episode, he was diagnosed with syphilis and treated with intramuscular penicillin G benzathine.

### 3.2 New capsular operon related to *Haemophilus sputorum* HSPU_type1

**Figure 1:**
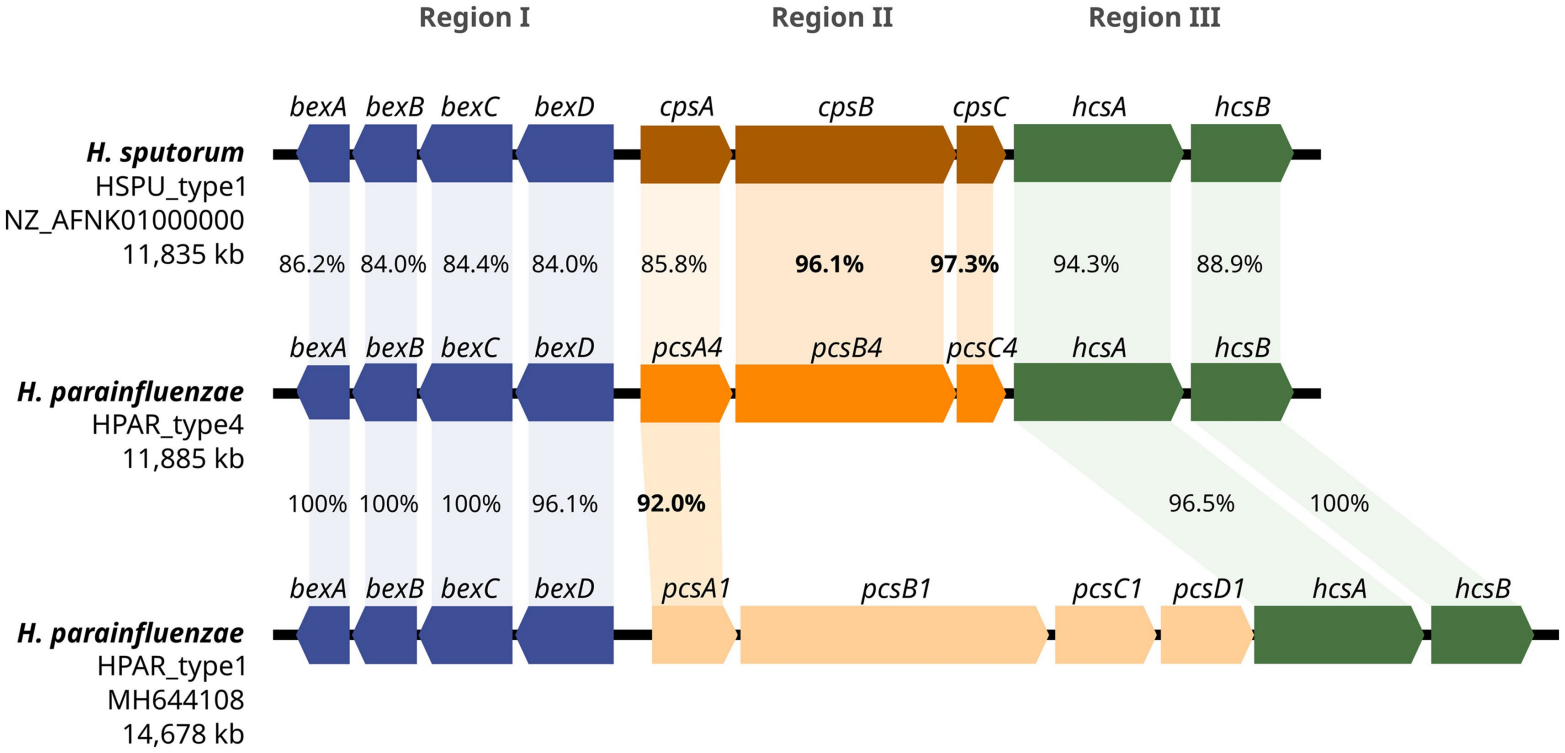
Genetic structure of the *H. parainfluenzae* HPAR_type4 operon. A comparison of the genetic organization between *H. parainfluenzae* HPAR_type4, *H. sputorum* HSPU_type1 and *H. parainfluenzae* HPAR_type1 operons is presented. The three main regions are represented using different colors, and the percentages of gene identity are displayed.

A novel capsular operon was identified by WGS as part of our hospital surveillance and was designated as HPAR_type4 (NCBI accession number PV567605). This operon was different from those previously described in *H. parainfluenzae*, with a total length of 11,885 bp and 9 predicted ORFs (Figure 1), following the typical genetic organization of *Haemophilus* spp. cap loci, comprising three regions (I, II, and III).

Regions I (*bexABCD*) and III (*hcsAB*) showed 96–100% homology to the corresponding regions in *H. parainfluenzae* HPAR_type1 (Supplementary Table S1). The detected ORFs were translated into protein sequences, with 98–100% identity to HPAR_type1. The serotype-specific region II contained three ORFs. The first ORF was homologous to the *pscA* gene found in *H. parainfluenzae* capsular operons, while the other two ORFs showed >96% identity to the *cpsB* and *cpsC* genes of *H. sputorum* HSPU_type1, encoding a glycosyltransferase and an acetyltransferase, respectively. We designated these genes as *pscA4* (1,101 bp), *pcsB4* (2,628 bp), and *pcsC4* (621 bp).

### 3.3 Antimicrobial resistance

Both strains exhibited an extensively drug-resistant phenotype. HUB-HP17268 was resistant to all tested β-lactams except ceftriaxone and carbapenems (imipenem and meropenem), whereas HUMT-HP05 was resistant to all tested β-lactams except for the carbapenems. Both strains were also resistant to macrolides, fluoroquinolones, chloramphenicol, trimethoprim-sulfamethoxazole and tetracycline (Table 1).

**Table 1:** MICs and molecular resistance mechanisms of *H. parainfluenzae* HPAR_type4 strains.

Isolate	β-lactams									
	AMP	AMC	CTX	CXM	FEP	CRO	IPM	MEM	Acquired genes	Substitutions in PBP3
HUB-HP17268	**4**	**4/2**	**0.25**	**>8**	**1**	≤0.12	1	≤0.25	–	I442F, V511A, N526K, V5621I
HUMT-HP05	**>4**	**4/2**	**>2**	**>8**	**>2**	**>0.5**	0.5	≤0.25	*bla_CTX-M-15_* *bla_TEM-1_*	S385T, I442F, V511A, N526K, V5621I

Isolate	Macrolides				Fluoroquinolones					
	ERY	AZM	Acquired genes	AcrB	NAL	CIP	LVX	MOX	GyrA	ParC
HUB-HP17268	**>16**	**>4**	*mef(A) msr(D)*	R321S	**>4**	**1**	**2**	**>2**	S84F, D88Y	S138T, M198L
HUMT-HP05	**>16**	**>4**	*mef(A) msr(D)*	–	**>4**	**1**	**1**	**>2**	S84F, D88Y	S138T, M198L

Isolate	Chloramphenicol		Cotrimoxazole					Tetracycline	
	CHL	Acquired genes	SXT	Acquired genes	FolA	FolA promoter	FolP	TET	Acquired genes
HUB-HP17268	**>8**	*catS*	**>2/38**	–	–	−10 motif G→A	–	**>4**	*tet(M)*
HUMT-HP05	**>8**	*catS*	**>2/38**	–	F154S	−10 motif G→A	–	**16**	*tet(M)*

Resistance interpretation of MICs according to the EUCAST criteria is marked in bold (see footnote 1). Data are shown as a range of values for microdilution (μg/ml).

AMP, ampicillin; AMC, amoxicillin/clavulanic acid; CTX, cefotaxime; CXM, cefuroxime; FEP, cefepime; CRO, ceftriaxone; IPM, imipenem; MEM, meropenem; ERY, erythromycin; AZM, azithromycin; NAL, nalidixic acid; CIP, ciprofloxacin; LVX, levofloxacin; MOX, moxifloxacin; CHL, chloramphenicol; SXT, trimethoprim-sulfamethoxazole; TET, tetracycline.

To further characterize the resistance mechanisms, we analyzed the genetic determinants in the genomes of both strains. Resistance to ceftriaxone in HUMT-HP05 led to the identification of the recently described CTX-M-15 extended spectrum β-lactamase, which was responsible for the high minimum inhibitory concentrations (MICs) for cephalosporins. The *bla*_CTX-M-15_ gene was inserted in a mobile element, showing >99% identity with the previously described ICE*HpaHUB6*. This strain also had a TEM-1 and resistance-associated modifications in PBP3 (i.e., S385T, I442F, V511A, N526K, V5621I) that contribute to β-lactam resistance. In HUB-HP17268, β-lactam resistance was only attributed to modifications in PBP3. Both strains had fluoroquinolones resistance caused by amino acid substitutions in GyrA (S84F and D88Y) and ParC (S138T and M198L). Macrolide and tetracycline resistance was conferred by the insertion of the *tet(M)-MEGA* element carrying the *mef*(E), *msr*(D) and *tet*(M) genes. Chloramphenicol resistance was due to the acquisition of *catS*, and trimethoprim-sulfamethoxazole resistance was attributed to mutations in the *folA* promoter (−10 motif [G > A]).

### 3.4 Genomic comparison

The genome of HUMT-HP05 consisted of 2,213,506 base pairs (bp) with a GC content of 39.5%, while the genome of HUB-HP17268 was slightly smaller, with 2,155,329 bp and a GC content of 39.6%. A total of 2,090,090 bp were shared between both genomes (94.4%). Within these shared regions, 17 recombinant blocks were identified, and only 42 SNPs were detected outside of these blocks. The presence of the ICE*HpaHUB6* mobile genetic element in HUMT-HP05 accounted for 58,725 bp of the genomic differences.

### 3.5 Plasmid characterization

**Figure 2:**
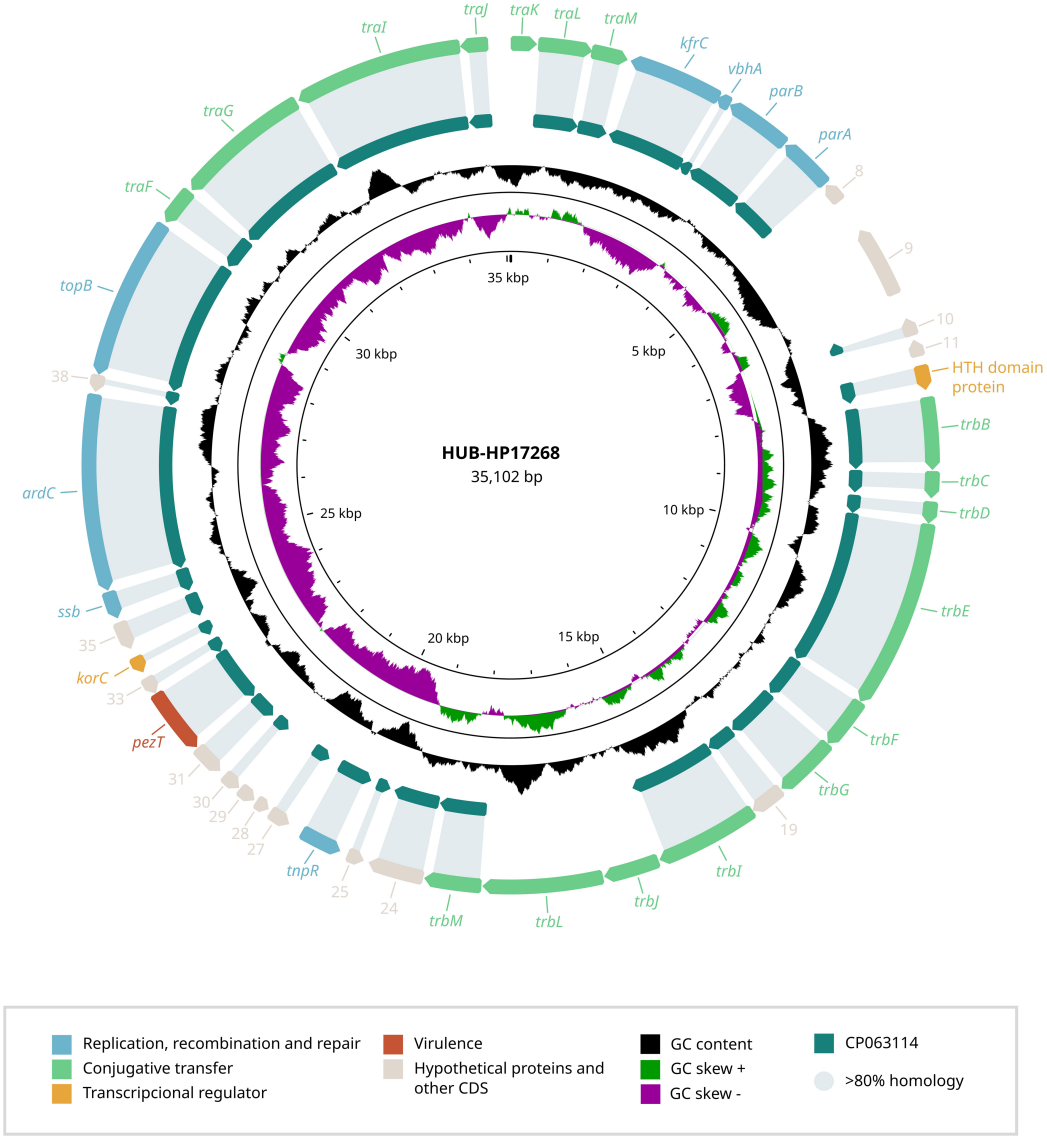
Genetic structure of the plasmid identified in *H. parainfluenzae* HUB-HP17268. The genetic organization of the HUB-HP17268 plasmid is shown, highlighting ORFs associated with replication, recombination and repair, conjugative transfer, transcriptional regulation, virulence, and hypothetical proteins. The GC content is also presented. A comparison with the most closely related plasmid found in *H. parainfluenzae* strain M1C147 (CP063114.1) is provided, displaying the percentage of gene identity.

**Figure 3:**
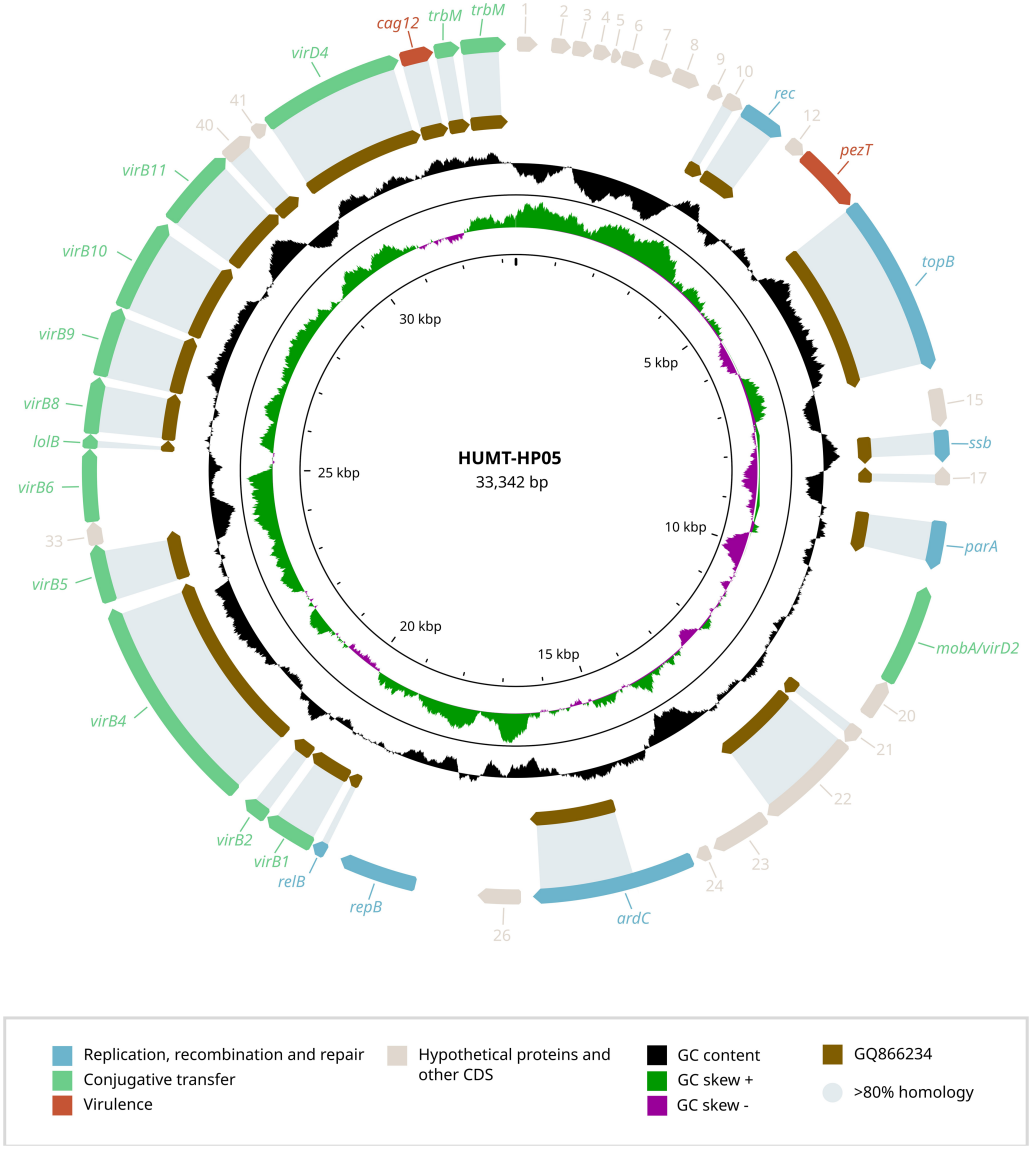
Genetic structure of the plasmid identified in *H. parainfluenzae* HUMT-HP05. The genetic organization of the HUMT-HP05 plasmid is shown, highlighting ORFs associated with replication, recombination and repair, conjugative transfer, virulence, and hypothetical proteins. The GC content is also presented. A comparison with the most closely related plasmid found in *A. actinomycetemcomitans* strain D11S-1 (GQ866234.1) is provided, displaying the percentage of gene identity.

In addition to the novel capsular operon, each strain carried a plasmid, previously unreported in *H. parainfluenzae* (Figures 2, 3) that was undetected by PlasmidFinder but identified through long-read sequencing.

To better understand these genetic elements, we conducted BLAST analyses of the plasmid sequences and their predicted ORFs against the NCBI public database, aiming to identify potential homologs and functional annotations. The plasmid carried by HUB-HP17268 (NCBI accession number PV567603) was 35,102 bp in length with a GC content of 33.0% and shared 89.0% identity with a plasmid from *H. parainfluenzae* strain M1C147 (CP063114.1). In contrast, HUMT-HP05 (NCBI accession number PV567604) harbored a plasmid of 33,342 bp with a GC content of 32.8%, which showed 89.1% homology with plasmid S25 from *Aggregatibacter actinomycetemcomitans* strain D11S-1 (GQ866234.1).

The complete annotation of predicted ORFs from both plasmids (Supplementary Table S2) identified genes strongly associated with plasmid replication, recombination and repair, including *repA* (replication protein), *parA* and *parB* (partitioning proteins), *topB* (DNA topoisomerase), and *ssb* (single-stranded DNA-binding protein). Neither of the plasmids contained resistance genes, but both harbored virulence-associated genes, such as *pezT*.

## 4 Discussion

The identification of a novel capsular operon, HPAR_type4, in two *H. parainfluenzae* strains isolated from genital ulcers in MSM patients expands our understanding of the genetic diversity of this species. This capsular operon was identified in two *H. parainfluenzae* strains isolated from different hospitals, with the episodes occurring 2 years apart. While there is no evidence suggesting direct contact between the patients, which initially appears to exclude a direct epidemiological link, the genetic similarity between the strains, sharing 96.7% homology, raises questions about potential common sources or transmission pathways. The absence of a standardized clonal classification system for *H. parainfluenzae* complicates the assessment of the epidemiological relationship between these strains. Further investigation is needed to determine if these cases represent an emerging trend or are isolated events.

Continuous genomic monitoring led to the detection of the first capsular operon in *H. parainfluenzae* (González-Díaz et al., 2019). Five years after its publication, four different capsular operons have already been found in this species (González-Díaz et al., 2019; Sierra et al., 2021; Bajanca-Lavado et al., 2022), including the HPAR_type4 operon described in this study. The presence of capsules in this genus may enhance their ability to evade host immune responses, as demonstrated in *H. influenzae*, where capsulated strains often exhibit increased pathogenicity (Guellil et al., 2022). Although the capsular operons found in *H. parainfluenzae* differ structurally from the virulent *H. influenzae* type b capsule, the notable capacity of this species to acquire new genetic material raises concerns about the potential emergence of similarly virulent capsules in the future (Sierra et al., 2021). Thus, its surveillance is important to monitor and respond to any emerging virulence factors.

The genetic organization of the capsular operon identified in HUB-HP17268 and HUMT-HP05 aligns with other *Haemophilus* spp. capsular loci, comprising three regions. The high homology of regions I and III with the previously characterized *H. parainfluenzae* HPAR_type1 operon (González-Díaz et al., 2019), indicates a conserved mechanism for capsular polysaccharide biosynthesis and export within the genus. The serotype-specific region II contains ORFs similar to genes from both *H. parainfluenzae* HPAR_type1 (*pcsA*) and *H. sputorum* HSPU_type1 (*cpsB* and *cpsC*), suggesting that horizontal gene transfer and recombination may play a role in the evolution of capsular diversity among *Haemophilus* species (Nielsen et al., 2015). Both strains were isolated from distinct Catalan towns over a 2-year period (without epidemiological connections), which excludes definitive conclusions about clonal transmission. However, the 17 recombinant blocks and the acquisition of CTX-M-15-bearing ICE*HpaHUB6* in HUMT-HP05 demonstrate the capacity of *H. parainfluenzae* for genomic recombination.

Since the first report of a capsular *H. parainfluenzae* strain, we have observed an alarming increase in antibiotic resistance. Both strains in our study exhibited XDR profiles, including resistance to β-lactams and other broad-spectrum antibiotics. This trend is consistent with our previous observations of capsulated MDR and XDR strains (Sierra et al., 2021). Interestingly, the first XDR strain described in the literature also carried an HPAR_type1 operon. We were able to identify this operon through genomic analysis of the sequence deposited in NCBI, as this detail was not reported in the original article (Tinguely et al., 2013). Since then, ESBL production has also been identified in this species. Initially, the *bla*_CTX-M-15_ gene was detected in four strains at our hospital (Saiz-Escobedo et al., 2023). Subsequently, another strain (ASM3628892v1) carrying the same resistance determinant was identified in France, with the particularity that this strain also carried the *H. parainfluenzae* HPAR_type1 operon (Caméléna et al., 2024). The identification of the CTX-M-15 ESBL in HUMT-HP05 highlights the ability of *H. parainfluenzae* to acquire resistance genes through mobile genetic elements (Juhas et al., 2007). The insertion of *bla*_CTX-M-15_ into the ICE*HpaHUB6* element may facilitate the spread of this resistance determinant within the species and among other bacteria co-colonizing the urogenital tract. Its detection in an MSM patient further underscores the importance of considering this pathogen in the context of STI management, especially in cases of co-infection with other pathogens, as it may serve as a reservoir for resistance genes.

To date, only transposons and ICE structures had been detected integrated into the *H. parainfluenzae* genome (Sierra et al., 2021). This study identified two previously unreported plasmids, both carrying genes essential for plasmid maintenance, replication and segregation, indicating their potential for propagation within host cells (Smillie et al., 2010). Among them, we identified a plasmid in *H. parainfluenzae* with significant homology to a plasmid from *A. actinomycetemcomitans*, a member of the same *Pasteurellaceae* family. This finding highlights the potential for horizontal transfer within this taxonomic group, which could contribute to the spread of adaptive traits, including antimicrobial resistance. Horizontal gene transfer is common in *Pasteurellaceae*, and aligns with recent mobilome analysis, which highlights the role of mobile genetic elements (including plasmids, transposons and integrons) in shaping the resistome of these pathogens (da Silva et al., 2022). Although none of the plasmids carried antibiotic resistance genes, unlike many clinically relevant plasmids (Watts et al., 2021), this does not preclude their potential to acquire such genes. Recently, several small plasmids have been detected in *Haemophilus* spp., carrying genes coding for the β-lactamases TEM-1 and TEM-40 (Watts et al., 2021). In this case, our strains were already XDR and had all the resistance determinants integrated in the genome (even the β-lactamase TEM-1 in the case of HUMT-HP05). However, the presence of these plasmids may help transfer these resistance determinants to other bacterial strains. Antimicrobial resistance genes can be incorporated through recombination events, such as the insertion of transposable elements (insertion sequences, transposons) or integrons carrying resistance determinants (Carattoli, 2013). The presence of recombination-associated genes on these plasmids suggest they have the machinery to facilitate such genetic exchanges, consistent with the role of plasmids as genetic reservoirs that can acquire and disseminate resistance genes under selective pressures (Carattoli, 2013).

In conclusion, this study highlights the genetic diversity of *H. parainfluenzae* and the emergence of multidrug-resistant strains in urogenital infections. The identification of new capsular operons enhancing virulence in XDR isolates, and the acquisition of resistance genes like the *bla*_CTX-M-15_, underscores the evolving threat of this pathogen. While these genomic findings are compelling, functional studies are needed to confirm the contribution of these capsular operons to pathogenicity. Nevertheless, these findings raise concerns about transmission, particularly in MSM populations, and emphasize the need for ongoing genomic surveillance to monitor the spread and pathogenic potential of these strains.

## Data Availability

The datasets presented in this study can be found in online repositories. The names of the repository/repositories and accession number(s) can be found in the article/Supplementary material.

## Footnotes

^1^ www.eucast.org/clinical_breakpoints

^2^ github.com/bactopia/bactopia

^3^ github.com/rrwick/Unicycler

^4^ github.com/tseemann/snippy

^5^ github.com/scwatts/hicap

^6^ github.com/ncbi/amr

^7^ cge.food.dtu.dk/services/PlasmidFinder/

^8^ github.com/tseemann/prokka

^9^ inkscape.org
