# Supplementary material for: A novel capsular operon and potentially conjugative plasmids in extensively drug-resistant urogenital Haemophilus parainfluenzae
Source: Front Microbiol. 2025 Oct 8;16:1659538. doi: 10.3389/fmicb.2025.1659538 (PMC12540396; doi:10.3389/fmicb.2025.1659538)
Supplement: Supplementary file 1 [file Table_1.DOCX]

**Table S1. Genetic identity of the *H. parainfluenzae* HPAR_type4 capsular operon.** A). The percentage of homology detected in each gene of the *H. parainfluenzae* HPAR_type4 operon concerning the capsular operons of *H. sputorum* HSPU_type1 and *H. parainfluenzae* HPAR_type1 is expressed. B). The percentage of homology between capsular operons detected in each protein is indicated. Gene length is presented in base pairs (bp) and protein length in the number of aminoacids (aa). The symbol '-' denotes the absence of a gene in the operon.

|  | *bexA* | | *bexB* | | *bexC* | | *bexD* | | *xcsA* | | *xcsB* | | *xcsC* | | *hcsA* | | *hcsB* | |
| --- | --- | --- | --- | --- | --- | --- | --- | --- | --- | --- | --- | --- | --- | --- | --- | --- | --- | --- |
| A | Identity (%) | Length (bp) | Identity (%) | Length (bp) | Identity (%) | Length (bp) | Identity (%) | Length (bp) | Identity (%) | Length (bp) | Identity (%) | Length (bp) | Identity (%) | Length (bp) | Identity (%) | Length (bp) | Identity (%) | Length (bp) |
| HPAR_type4 |  | 654 |  | 798 |  | 1134 |  | 1260 |  | 1101 |  | 2628 |  | 621 |  | 2043 |  | 1221 |
| HPAR_type1 | 100 | 654 | 100 | 798 | 100 | 1134 | 96.1 | 1185 | 92.0 | 939 | 50.6 | 3717 | 52.3 | 1230 | 96.5 | 2043 | 100 | 1221 |
| HSPU_type1 | 86.2 | 654 | 84.0 | 798 | 84.4 | 1134 | 84.0 | 1185 | 85.8 | 1101 | 96.1 | 2628 | 97.3 | 621 | 94.3 | 2043 | 88.9 | 1221 |

|  | BexA | | BexB | | BexC | | BexD | | XcsA | | XcsB | | XcsC | | HcsA | | HcsB | |
| --- | --- | --- | --- | --- | --- | --- | --- | --- | --- | --- | --- | --- | --- | --- | --- | --- | --- | --- |
| B | Identity (%) | Length (aa) | Identity (%) | Length (aa) | Identity (%) | Length (aa) | Identity (%) | Length (aa) | Identity (%) | Length (aa) | Identity (%) | Length (aa) | Identity (%) | Length (aa) | Identity (%) | Length (aa) | Identity (%) | Length (aa) |
| HPAR_type4 |  | 217 |  | 265 |  | 377 |  | 420 |  | 366 |  | 875 |  | 206 |  | 680 |  | 406 |
| HPAR_type1 | 100 | 217 | 100 | 265 | 100 | 377 | 90.1 | 394 | 93.3 | 312 | 32.3 | 1238 | 25.0 | 409 | 97.8 | 680 | 100 | 406 |
| HSPU_type1 | 98.2 | 217 | 94.0 | 265 | 90.2 | 377 | 91.6 | 394 | 89.1 | 366 | 96.6 | 875 | 93.2 | 206 | 94.7 | 680 | 90.1 | 406 |
